# 

**Published:** 1899-08-19

**Authors:** 


					s\ ^ THE HOSPITAL. " The standard of highest purity?Lancet. August 19th, 1899 '
CADBURY'S i^-Vf ^ CA DBU
COCOA JL MM COCOA ^
ABSOLUTELY PURS. NO DRUGS OR ALKALIES USED. S. C. (,_
/scow WfSTeflw Infirmary
No. 673T* Vol. XXVI. GfS?3?] Satordat,
August 19th, 1899.
[SabsoriptionTems.J pB(ICE TWOPENCE.

				

